# On the road to ‘research municipalities’: analysing transdisciplinarity in municipal ecosystem services and adaptation planning

**DOI:** 10.1007/s11625-017-0499-0

**Published:** 2017-11-16

**Authors:** Ebba Brink, Christine Wamsler, Maria Adolfsson, Monica Axelsson, Thomas Beery, Helena Björn, Torleif Bramryd, Nils Ekelund, Therese Jephson, Widar Narvelo, Barry Ness, K. Ingemar Jönsson, Thomas Palo, Magnus Sjeldrup, Sanna Stålhammar, Geraldine Thiere

**Affiliations:** 1 Lund University Centre for Sustainability Studies (LUCSUS), P.O. Box 170, 221 00 Lund, Sweden; 2 Lund University Centre of Excellence for Integration of Social and Natural Dimensions of Sustainability (LUCID), P.O. Box 170, 221 00 Lund, Sweden; 3 Department of Sustainable Development, Trelleborg Municipality, Algatan 13, 231 83 Trelleborg, Sweden; 4 Department for Environment and Urban Planning, Kristianstad Municipality, 291 80 Kristianstad, Sweden; 5 School of Education and Environment, Kristianstad University, 291 88 Kristianstad, Sweden; 6 Minnesota Sea Grant, University of Minnesota Duluth, 31 W College St, Duluth, MN 55812 USA; 7 Department of Sustainable Development (Planning Section), Lomma Municipality, 234 81 Lomma, Sweden; 8 Environmental Strategy, ISM, Lund University, Campus Helsingborg, P.O. Box 882, 251 08 Helsingborg, Sweden; 9 Department of Science, Environment, Society, Malmö University, 205 06 Malmö, Sweden; 10 Research and Development, Scania Association of Local Authorities (SALA), Box 53, 221 00 Lund, Sweden; 11 Comprehensive Planning Unit, City Planning and Technical Services Department, Helsingborg municipality, 251 89 Helsingborg, Sweden; 12 Department of Wildlife, Fish and Environmental Studies, SLU Umeå, 901 83 Umeå, Sweden; 13 City Planning Department, Bjuv Municipality, Box 501, 267 25 Bjuv, Sweden

**Keywords:** Transdisciplinarity, Ecosystem services, Project assessment, Collaborative sustainability research, Sweden, Urban planning

## Abstract

Transdisciplinary research and collaboration is widely acknowledged as a critical success factor for solution-oriented approaches that can tackle complex sustainability challenges, such as biodiversity loss, pollution, and climate-related hazards. In this context, city governments’ engagement in transdisciplinarity is generally seen as a key condition for societal transformation towards sustainability. However, empirical evidence is rare. This paper presents a self-assessment of a joint research project on ecosystem services and climate adaptation planning (ECOSIMP) undertaken by four universities and seven Swedish municipalities. We apply a set of design principles and guiding questions for transdisciplinary sustainability projects and, on this basis, identify key aspects for supporting university–municipality collaboration. We show that: (1) selecting the number and type of project stakeholders requires more explicit consideration of the purpose of societal actors’ participation; (2) concrete, interim benefits for participating practitioners and organisations need to be continuously discussed; (3) promoting the ‘inter’, i.e., interdisciplinary and inter-city learning, can support transdisciplinarity and, ultimately, urban sustainability and long-term change. In this context, we found that design principles for transdisciplinarity have the potential to (4) mitigate project shortcomings, even when transdisciplinarity is not an explicit aim, and (5) address differences and allow new voices to be heard. We propose additional guiding questions to address shortcomings and inspire reflexivity in transdisciplinary projects.

## Introduction

Transdisciplinarity is promoted as a solution-oriented research approach for addressing complex sustainability challenges (Brandt et al. 2013; Hirsch Hadorn et al. 2006; Lang et al. 2012; Wiek et al. 2012) such as biodiversity loss, pollution and climate-related hazards. Although there are diverging interpretations of transdisciplinarity (e.g., Max-Neef 2005), most advocates agree that it is characterised by: (1) complex societal problems (often involving multiple interests and interacting challenges); (2) collaboration between and among scientific disciplines and societal actors; and (3) processes of mutual learning between science and society for joint problem-solving (Brandt et al. 2013; Jahn et al. 2012). Closely related to concepts such as participatory action research (Glassman and Erdem 2014; Streck 2014) and post-normal science (Funtowicz and Ravetz 1993), it is argued that transdisciplinarity is necessary for effective science and societal change as it can help uncover underlying assumptions in research and practice, and develop methodologies for working with uncertainties and disputed values (Jahn et al. 2012; Lang et al. 2012).

Accordingly, transdisciplinary research, collaboration, and learning are seen as crucial for achieving urban transformation towards sustainability (McCormick et al. 2013). Transformation, here, refers to a deliberate (while not fully steerable) process of structural change in a normative direction (Feola 2014). In this context, municipalities are seen as key actors (Roberts 2008; SALA 2016a; Statskontoret 2016). Their influence on urban planning as well as their vast experience in place-based environmental problem-solving and mediation with other stakeholders (Bulkeley and Betsill 2005) makes them key collaboration partners for transdisciplinary sustainability research (see Wiek et al. 2012).

While research and governance are becoming increasingly intertwined in the pursuit of urban sustainability, empirical analyses of transdisciplinary approaches that can (or cannot) produce actionable and rigorous results (i.e., results that are useful to social actors and satisfy scientific quality criteria) are rare. However, such analyses are crucial, since collaboration with, or co-funding by, societal actors is increasingly required for securing research funding in the planning and environmental sciences (Jahn et al. 2012). Past studies have pointed towards general challenges, such as differences in professional cultures and rationale between scientists and planners (Ahern et al. 2014; Polk 2014), power asymmetries between participants (Jahn et al. 2012; Mobjörk 2010; Wittmayer and Schäpke 2014), and the incompatibility of transdisciplinary projects or findings with traditional (academic or municipal) institutional structures (Brandt et al. 2013; Polk 2014; Wiek et al. 2012).

Against this background, the aim of this paper is to assess, through a transdisciplinarity lens, a sustainability research project on ecosystem services planning and climate adaptation, carried out in collaboration with seven Swedish municipalities. The ecosystem services concept denotes the “conditions and processes through which natural ecosystems […] sustain and fulfil human life” (Daily 1997, p. 3). In this paper, ecosystem services planning describes a place-based approach that focuses on the creation, restoration, and conservation of ecological structures to provide society with specific services from nature (Chan et al. 2006; Staes et al. 2010), while climate adaptation (or ‘adaptation’ in short) is “the process of adjustment to actual or expected climate and its effects” (IPCC 2014, p. 1758).

We use a participatory case study methodology (Scholz et al. 2006; Yin 2008) to self-assess the project ‘Implementing the Ecosystem Services Concept at the Municipal Level’ (ECOSIMP 2013–2017). ECOSIMP was based on a general idea of transdisciplinarity as research collaboration with actors outside academia (which we hereafter shall refer to as participatory research^1^). ECOSIMP was not, however, structured around academic principles of transdisciplinarity, nor did it have transdisciplinarity as an explicit aim or success criteria. In this paper, we apply the design principles for transdisciplinarity created by Lang et al. (2012)—often considered to represent the ‘state of the art’ of transdisciplinary research—to assess with project stakeholders: (1) how ECOSIMP has approached and delivered on its transdisciplinary potential and (2) what lessons can be learned for the design and assessment of similar research collaborations.

The following section (“Analysis framework”) describes the framework used for the assessment, before we introduce our methodology, including the project description and rationale (“Methodology”). “Results” presents the assessment of the project’s design and implementation against prescribed phases for transdisciplinary projects. Next, we discuss the lessons learned from the appraisal and reflect on the use of the design principles (“Discussion”), before we summarise our contribution in “Conclusion”.

## Analysis framework

**Fig. 1:**
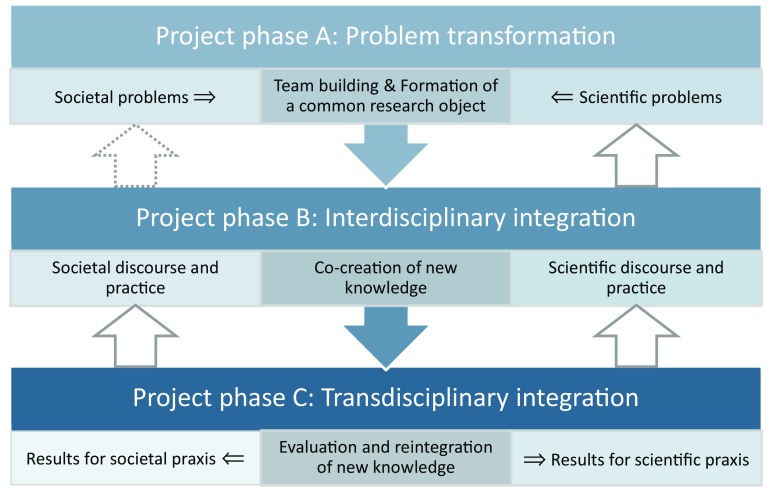
Conceptual model of an ideal–typical transdisciplinary project (adapted from Lang et al., 2012; Jahn et al. 2012)

Transdisciplinary projects can be conceptualised in terms of the following three phases: problem transformation (project phase A), interdisciplinary integration (project phase B), and transdisciplinary integration (project phase C) (see Fig. 1) (Jahn et al. 2012; Lang et al. 2012; see also, e.g., Hirsch Hadorn and Pohl 2007; Talwar et al. 2011 for similar project representations). As shown in Fig. 1, this creates opportunities to conceptualise and assess transdisciplinary contributions to societal and scientific progress as “two epistemic ends of the same research dynamic” (Jahn et al. 2012, p. 4), illustrated by the horizontal arrows in phase C. In simple terms, this means that addressing real-world problems should be combined with addressing gaps in scientific knowledge.

Each phase (A–C) of a transdisciplinary project is characterised by specific actions prescribed by design principles (Table 1).

**Table 1:** Design principles and guiding questions for transdisciplinary research projects (Lang et al. 2012) adapted to ECOSIMP

Design principle	Guiding question (Lang et al. 2012)	Adapted to ECOSIMP
Project phase A		
1. Build a collaborative research team	Does (did/will) the project team include all relevant expertise, experience, and other relevant ‘stakes’ needed to tackle the sustainability problem in a way that provides solution options and contributes to the related scientific body of knowledge?	Did the project team include all relevant expertise, experience, and other relevant ‘stakes’ needed to increase knowledge and provide the tools for the consideration of ecosystem services in municipal planning and climate adaptation?
2. Create joint understanding and definition of the sustainability problem to be addressed	Does the project team reach a common understanding of the sustainability problem to be addressed and does the team accept a joint definition of the problem?	Did the project team reach a common understanding of the ‘real-world’ sustainability problem to be addressed by the project, and was an explicit definition of this problem formulated and agreed on by all team members?
3. Collaboratively define the boundary/research object, research objectives as well as specific research questions, and success criteria	Is a common research object or guiding question, with subsequent specified research object[ive] and questions, formulated, and does the partners agree on common success criteria?	Did the project members agree on using the ecosystem services concept as a common research object, and were related research aims, questions and success criteria formulated, and agreed on by all team members?
4. Design a methodological framework for collaborative knowledge production and integration	Does the project team agree upon a jointly developed methodological framework that defines how the research target will be pursued in Phase B and what transdisciplinary settings will be employed? Does the framework adequately account for both the collaboration among the scientific fields and with the practice partners?	Did the project team agree upon a jointly developed methodological framework that defined how the research target should be pursued in phase B and what transdisciplinary settings should be employed? Did the project organisation adequately account for both collaboration among scientific fields and how/whether researchers should collaborate with the municipal representatives in between workshops?
Project phase B		
1. Appropriate roles for practitioners and researchers	Are the tasks and roles of the actors from science and practice involved in the research process clearly defined?	Were the tasks and roles of the involved researchers and municipality representatives clearly defined?
2. Apply and adjust integrative research methods and transdisciplinary settings for knowledge generation and integration	Does the research team employ or develop methods suitable to generate solution options for the problem addressed? Does the team employ or develop suitable settings for inter- and transdisciplinary cooperation and knowledge integration?	Did the team employ or develop suitable settings for inter- and transdisciplinary cooperation and knowledge integration? Did the research team employ or develop methods suitable to generate solution options to address the lack of knowledge and tools for consideration of ecosystem services in municipal planning and climate adaptation?
Project phase C		
1. Realize two-dimensional integration	Are the project results implemented to resolve or mitigate the problem addressed? Are the results integrated into the existing scientific body of knowledge for transfer and scaling-up efforts?	Have the project’s results been implemented to resolve or mitigate the lack of knowledge and tools for ecosystem service consideration in municipal planning? Were the results integrated into the existing scientific body of knowledge for transfer and scaling-up efforts?
2. Generate targeted ‘‘products’’ for both parties	Does the research team provide practice partners and scientists with products, publications, services, etc. in an appropriate form and language?	Did ECOSIMP provide Swedish-language tools, guidelines, pamphlets, reports, or other products useful for municipalities? Were peer-reviewed articles, book chapters and conference presentations generated in Swedish and English?
3. Evaluate scientific and societal impact	Are the goals being achieved? What additional (unanticipated) positive effects are being accomplished?	Were the goals achieved? What additional (unanticipated) positive effects were created?
Cross-cutting principles (X)		
1. Facilitate continuous formative evaluation	Is a formative evaluation being conducted involving relevant experts related to the topical field and transdisciplinary research (throughout the project)?	Has formative evaluation been conducted throughout the project, involving relevant experts on the empirical content (ecosystem services, climate adaptation and municipal planning) as well as transdisciplinarity?
2. Mitigate conflict constellations	Do the researchers/practitioners prepare for/anticipate conflict at the outset, and are procedures/processes being adopted for managing conflict as and when it arises?	Did the researchers and municipality representatives prepare for/anticipate conflict at the outset, and were procedures/processes adopted for managing conflict as and when it arose?
3. Enhance capabilities for and interest in participation	Is adequate attention being paid to the (material and intellectual) capabilities that are required for effective and sustained participation in the project over time?	Has adequate attention been paid to the (material and intellectual) capabilities required for effective and sustained participation in the project over time?

### Phase A: problem transformation

The problem transformation phase (phase A) consists of team building, creating a common definition of the (societal and related scientific) problem to be addressed, formulating project aims, research questions and success criteria, and creating a framework for collaboration. The latter entails participants agreeing on methods and transdisciplinary settings, and developing a concept or framework for integrating the project’s results throughout its course (Lang et al. 2012).

### Phase B: interdisciplinary integration

The interdisciplinary integration phase (phase B) is where the actual research, or co-creation of knowledge, occurs. It includes assigning appropriate roles to researchers and societal actors, and adopting (and potentially further developing) research methods to support the integration of knowledge held by the different participants. This phase may require different levels of stakeholder involvement (Lang et al. 2012), and most scholars would agree that a transdisciplinary approach also entails disciplinary work (Jahn et al. 2012). Nevertheless, the primary operation by which new knowledge is created is described as integration. Integration comprises linking and demarcating bodies of knowledge (epistemic integration), clarifying and mediating between the goals of different project components and actors (social-organisational integration) and establishing common vocabulary (communicative integration) in novel ways in the given problem context (Bunders et al. 2010; Jahn et al. 2012).

### Phase C: transdisciplinary integration

The transdisciplinary integration phase (phase C) involves the project participants’ evaluation of the co-created knowledge against both scientific and societal criteria, implementation of the knowledge, and, ultimately, an evaluation of the project’s (scientific and societal) impact. In contrast to the traditional science–policy transfer, this phase entails the (re)integration of knowledge from the transdisciplinary learning space into both societal and scientific practices (Lang et al. 2012). In the case of societal practice, this can refer to strategies or action programmes generated during the research process; however, there are also less-tangible outcomes for societal stakeholders, such as enhanced capacity, motivation, and feelings of ownership for the strategies that have been created (Lang et al. 2012).

### Cross-cutting principles

Equally important, are a set of cross-cutting principles (represented as X in Table 1 and “Results”) that should be considered during all three project phases. These principles include continuous formative project evaluation, proactive conflict management, and enhancing the (material and intellectual) capabilities for participation (Lang et al. 2012). While the model in Fig. 1 is conceptualised as a linear progression through the project phases, in practice, many transdisciplinary processes are iterative and may require revisiting phase B or even phase A (Jahn et al. 2012; Lang et al. 2012).

## Methodology

### The ECOSIMP project—case description and rationale

The ECOSIMP project (2013–2017) focused on the implementation of the ecosystem services concept in Swedish municipal planning and associated ecosystem-based adaptation to climate-related hazards. ECOSIMP was one of seven projects funded by the Swedish Environmental Protection Agency (SEPA) under the ‘Value of ecosystem services’ initiative (http://www.ecosystemservices.se/). The research initiative was motivated by an Interim Target to fulfil the national environmental quality objectives. Established in 2012, the Interim Target requires the importance of biodiversity and the value of ecosystem services to be widely-known, and integrated into economic and political considerations and societal decisions by 2018 (Borgström 2013; SEPA 2012). While several of the other projects funded through the initiative focused explicitly on ecosystem services valuation, ECOSIMP took a complementary approach by examining the extent to which institutional and cultural conditions have allowed the consideration of ecosystem services to influence municipal decisions (Jönsson et al. 2013). The ‘real-world’ sustainability problem that ECOSIMP targeted is defined in Box 1. An overview of ECOSIMP and its different subprojects is shown in Table 2.

**Table 2:** Overview over ECOSIMP subprojects, where *ES* denotes ecosystem services, and *EbA* ecosystem-based adaptation

Subproject	Aim of subproject	Data collection (Stakeholder involvement approach^a^)	Data analysis (Methods of knowledge integration)	References
A. Perceptions of the ES concept	Investigate municipal perceptions of the ES concept and its usefulness, to understand the basis for integration into planning and decision-making	Interviews with municipal planners and politicians, following consultation of ECOSIMP municipality representatives about interview participant selection and interview questions	Grounded theory and qualitative coding of interview transcripts	Beery et al. 2016
B. Obstacles and opportunities for ES implementation	Investigate municipal perceptions of barriers and opportunities of ES implementation in municipal planning and decision-making	Use of data from A. Feedback rounds with municipality representatives	Grounded theory and qualitative coding of interview transcripts	Beery et al. 2016
C. ES in comprehensive and detailed planning	Analyse explicit and implicit use of ES in comprehensive plans and review the scientific views on comprehensive plans as a tool for ES implementation	Review of comprehensive plans and citation statistics, interviews with planners in selected municipalities. Use of data from A	Grounded theory, qualitative and quantitative content analysis of comprehensive plans and interview data	Palo et al. 2016; Schubert et al. 2017a
D. Ecosystem-based adaptation (EbA)	Identify existing and potential ways and benefits of combining ES, EbA and climate adaptation in municipal planning and operations	Interviews, focus group discussions, participant observation and feedback rounds with municipality representatives. Some use of interview data from A	Grounded theory, systems theory, qualitative coding and analysis of interview transcripts	Brink and Wa msler 2017; Wamsler 2015b; Wamsler et al. 2014, 2016; Wamsler and Brink 2016
E. ES and transdisciplinarity	Evaluate and synthesise the experience of a transdisciplinary ES project with municipalities	Workshop discussions, participant observation, SWOT analysis, participant survey, ongoing dialogue and feedback rounds with project participants	Analysis of project documentation based on design principles for transdisciplinarity	(The present article)
F. Applied case studies of ES implementation (cross-cutting^b^)	Illustrate and analyse current ES-related problems and potential solutions in the municipal context	Interviews and focus groups with municipal planners. Review of municipal documents and local newspaper articles. Citizen focus groups and hearings	Different; depending on discipline and subproject aim (see above)	Bramryd and Johansson 2016; Schubert et al. 2017a

	Båstad case	Helsingborg case	Kristianstad case	Lomma case	Simrishamn case	Trelleborg case
	Identifies ES in five key periurban green spaces in Båstad. The resulting report is expected to be useful as a basis for planning and decision-making in Båstad municipality	Contrasts the ecological functions of the Görarp Pond with planned developments to reveal conflicts of interests that may arise with the current policy options	Analyses perceptions/value of nature in the history and development of the Härlöv landfill, and how its establishment and potential conversion into a recreation area affect ES	Studies the development of a Coastal Adaptation Strategy, including its stakeholder involvement, and the degree of mainstreaming of risk reduction and climate adaptation	Focuses on the Vitemölla nature reserve, and the conflict between a conservation plan that meant deforestation of invasive tree species and local residents’ preferences	Studies the trade-offs between valuable ES and road construction in the Dalköpinge river area, including characteristic biotopes and how existing ES can be enhanced

^a^ All subprojects benefited from regular workshops with municipality representatives

^b^ The focus of the case studies was primarily based on what the municipalities deemed useful; therefore, the level of integration/overlap between case studies and subprojects varied throughout the project

ECOSIMP’s geographical setting and thematic focus provided a rich context to investigate (principles of) transdisciplinarity. Sweden has a long history of decentralised environmental and climate policy work (SymbioCity 2011). The Scania region, the project’s geographical focus, has branded itself as a knowledge- and innovation-driven region that promotes university–municipality collaboration and making research more relevant and useful for municipalities (Lagercrantz and Palo 2012; SALA 2016a). An important factor that helped to create the conditions for ECOSIMP was a regional science–policy network on ecosystem services planning initiated by Scania’s Association for Local Authorities (SALA) in 2012. In addition, together with a local university, SALA has developed the ‘research municipality’ concept (*forskningskommun*) (SALA 2016b): a certification for Scanian municipalities that use participation in research as part of their strategic development work in environmental and urban planning.

Meanwhile, the ecosystem services concept has rapidly gained momentum in urban research and planning worldwide (Ahern et al. 2014; Gómez-Baggethun and Barton 2013; Luederitz et al. 2015; Niemelä et al. 2010; Woodruff and BenDor 2016) and is considered to be a promising tool for linking researchers and policy makers on a common sustainability agenda (Abson et al. 2014). However, the concept’s use and outcomes remain uncertain and contested (e.g., McCauley 2006; Turnhout et al. 2013) and there is a lack of practical knowledge on how to foster ecosystem services planning (Borgström 2013; Ernstson et al. 2010).

**Box 1:**
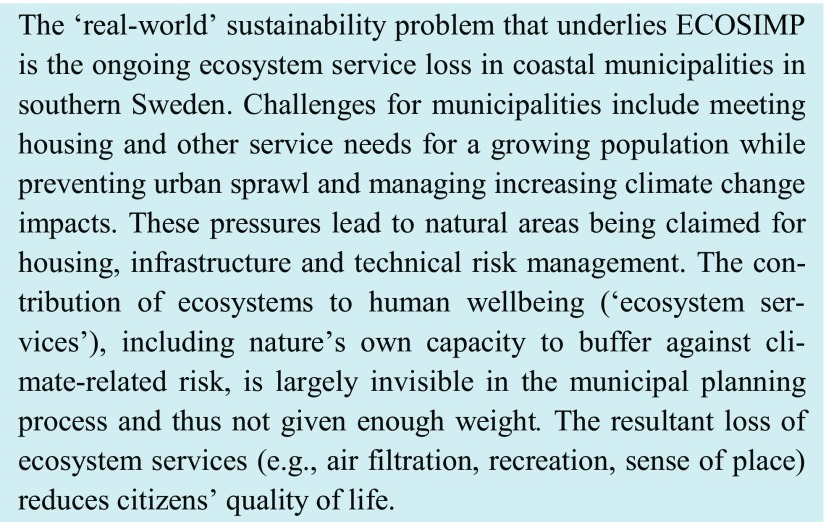
Sustainability problem

### Method for co-creating data

In contrast to ECOSIMP’s other subprojects (see Table 2), the study presented in this paper used routine project workshops, seminars, and meetings as the main arenas for data creation, collection, and validation. As is considered imperative for participatory research, we strived for an iterative, non-hierarchal dialogue in which all project participants (i.e., researchers and municipality representatives) were equally important problem solvers, thinkers and learners (Glassman and Erdem 2014). The research process followed the four, partly overlapping steps described below. Where the analysis was conducted or synthesised by a smaller group of researchers, iteration with and feedback from the larger project group was used to minimise any personal bias.

#### Step 1: Framework selection and modification

The idea to analyse the project using transdisciplinary principles was pitched to project participants in October 2015 by a participating PhD student in Sustainability Science. The design principles and guiding questions developed by Lang et al. (2012) and Jahn et al. (2012), which were previously not familiar to the group of senior researchers who designed the project, were hereby introduced to and discussed with all project participants, and modified to fit the context of the ECOSIMP project.

#### Step 2: Data collection/creation

Data primarily consisted of recordings, notes, and written observations produced by participants in project workshops, seminars, and meetings.^2^ Meetings where the ‘meta-issue’ of transdisciplinarity was explicitly discussed (as opposed to the empirical issues of ecosystem services and ecosystem-based adaptation) were particularly useful for this paper. These included a joint SWOT (Strengths, Weaknesses, Opportunities, and Threats) analysis of the participatory working method, conducted with 11 researchers and six municipality representatives. This was followed-up with a short, open-ended written questionnaire and a joint discussion during a workshop structured around the design principles for transdisciplinarity (with seven researchers and seven municipality representatives). Finally, official project reports (e.g., Jönsson et al. 2017; Palo, 2013) and project outputs (e.g., Beery et al. 2016; Wamsler et al. 2014) were used, mainly to support this article’s empirical description of the project (e.g., context, official aims, and results).

#### Step 3: Data coding

Data were coded based on the conceptual categories outlined in “Analysis framework”. Literal reading (Crabtree and Miller 1999) and content analysis (Mayring 2000) were applied (by one researcher) to extract and categorise evidence relevant to the transdisciplinary design principles, with regular input from, and checks by, the larger project group. Preliminary results, and their implications for ecosystem services planning, climate adaptation, and research–municipality collaboration, were jointly discussed during a project workshop in June 2016.

#### Step 4: Synthesis and writing

Finally, the revised results and resultant manuscript were subject to internal review by project participants in May–June 2016, and February 2017.

## Results

The results of the analysis are presented in terms of the three project phases (A, B, and C) and the cross-cutting principles (X) given in “Analysis framework”. For each principle, we provide an overall assessment of ECOSIMP’s attainment (low, medium or high).

### A1. Collaborative research team

- **Guiding question:** Did the project team include all relevant expertise, experience, and other relevant ‘stakes’ needed to increase knowledge and provide the tools for the consideration of ecosystem services in municipal planning and climate adaptation?
- **Attainment:** Medium

The ECOSIMP team (see Table 3) was made up of researchers and municipal civil servants (hereafter referred to as ‘municipality representatives’) drawn from a science–policy network on ecosystem services in municipal planning, initiated by SALA. Consequently, some participants had experience in acting at the interface of research and practice, including a series of roundtable discussions (‘research circles’) on planning under climate uncertainty, organised by SALA (see Palo 2013). The project’s team, however, mainly included new actors who did not have a history of collaboration. The initiative was seen as a form of regional pilot for university–municipality collaboration in environmental and urban planning. Project participants were from seven Swedish coastal municipalities: Båstad, Helsingborg, Kristianstad, Lomma, Malmö, Simrishamn, and Trelleborg, and four Swedish Universities. Researchers included PhD students, post-doctoral researchers, and professors. Municipality representatives, of whom several held a doctoral degree, were mainly ecologists, environmental strategists, and planners. Additional societal actors were SALA, the Marine Centre Simrishamn and the County Council (*Region Skåne)*.

**Table 3:** Overview of ECOSIMP’s participants (throughout the project’s lifetime)

ECOSIMP actor type	Organisation	Number of participants
Local authority	Båstad municipality	1
	Helsingborg city	1
	Kristianstad municipality	2
	Lomma municipality	3
	Malmö city	1
	Simrishamn municipality	2
	Trelleborg municipality	1
Regional organisation	Scanian Association for Local Authorities (SALA)	3
	Region Skåne (County Council)	2
Research group	School of Education and Environment; Kristianstad University	2
	Department of Science, Environment and Society; Malmö University	2
	Environmental Strategy, Department of Service Management and Service Studies; Lund University (Campus Helsingborg)	2
	Centre for Sustainability Studies; Lund University	3
	Department of Wildlife, Fish and Environmental Studies; Swedish University of Agricultural Sciences (SLU Umeå)	1
Total		26

Having a ‘stake’ in ecosystem services planning and climate adaptation can refer to actors that are responsible (typically municipalities), or those who are affected by the issue or its management (typically citizens and developers). The ECOSIMP project focused on municipal actors, based on this group’s legitimacy, accountability, and its ability to reach out to, and impact, both corporate decision makers and society at large (Jönsson et al. 2013). Participants agreed that the formal inclusion of other parties would have considerably increased the complexity and resource intensity of the project. Nevertheless, during phases A and B, the role of citizens in ecosystem services and adaptation planning was extensively discussed, due to: (1) the region’s high ratio of privately owned land; (2) the fact that citizens are impacted by municipal planning (which should be carried out in the public interest); and (3) their responsibilities with regard to climate-related hazards (Adger et al. 2013; Brink and Wamsler 2017; Wamsler and Brink 2014). Consequently, several project components and associated results integrate the views of citizens (Helsingborg and Lomma case studies, see Table 2 [subproject F] and Box 3).

The research team consisted of more researchers from a natural science, positivist tradition, and fewer social scientists. To create a better balance, researchers from sustainability science, who used predominantly qualitative methods, were added. No such action was taken for municipality representatives, which led to a lack of representation of the social divisions of municipal government in the project. However, both planners and ecologists were included. The higher proportion of ecologists (among both researchers and municipality representatives) can be attributed to the fact that the ecosystem services concept is often perceived as pertaining to ecology (despite also representing social benefits). In addition, since many municipal ecologists and planners perceive that ecology/ecological planning has low status (compared to, e.g., the promotion of economic growth or collaboration with industry), it was seen as counter-productive to include these more powerful interests in strategy development. Instead, politicians and other policy makers were involved through targeted activities (e.g., interviews, workshops) to support long-term change.

### A2. Sustainability problem

- **Guiding question:** Did the project team reach a common understanding of the ‘real-world’ sustainability problem to be addressed by the project, and was an explicit definition of this problem formulated and agreed on by all team members?
- **Attainment:** Low-Medium

The initial idea for the project followed an extensive interview-based survey of research needs in 24 Scanian municipalities carried out by their interest organisation SALA between 2010 and 2012 (Lagercrantz and Palo 2012). It identified topics that required intense research collaboration, including: comprehensive planning (in theory and practice); coastal zone management in relation with climate and development pressures; and enhancing human wellbeing while protecting the environment. In roundtable meetings following the survey, the ecosystem services concept was considered by several municipalities as an avenue for further exploration, which became the basis for ECOSIMP. Climate adaptation, notably tensions between technical and ecosystem-based approaches, was often the entry point into ecosystem services for municipalities, and it was, therefore, given special attention (Table 2).

Transdisciplinary design principles recommend a general definition of the real-life sustainability problem to be formulated, which is distinct from the scientific problem and agreed on by all project participants. In ECOSIMP, such a societal problem definition was explicitly formulated late in the final project stage, and for the purpose of this paper (see Box 1). Despite this apparent shortcoming, participants considered themselves to have a shared understanding of the sustainability problem(s) to be addressed, based on prior work carried out by SALA, and the resultant context-based scientific problem framing.

### A3. Boundary object, research objectives, and success criteria

- **Guiding question:** Did the project members agree on using the ecosystem services concept as a common research object, and were related research aims, questions and success criteria formulated, and agreed on by all team members?
- **Attainment:** Medium

The focus, objectives, and research questions (see Box 2) for the project were formulated by a group of five senior researchers who took responsibility for writing the research proposal, based on initial suggestions and feedback from municipalities (e.g., during previous SALA networking events). The overall objective was to analyse municipalities’ past decisions, current planning, and future challenges from an ecosystem services perspective to increase understanding of the ecosystem services concept (and associated ecosystem-based adaptation) as a tool for sustainable development (Jönsson et al. 2013).

**Box 2:**
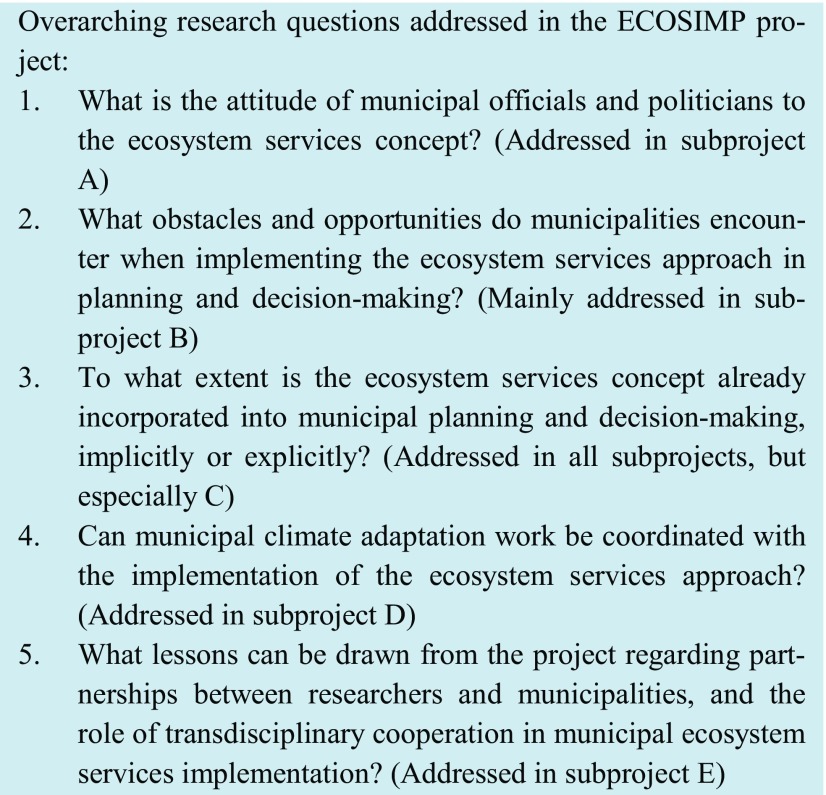
Scientific problem/research questions

The ecosystem services concept, whose use was required by SEPA in the call for proposals, worked as a boundary object by providing a shared framework for collaboration on very different societal and scientific issues relevant to sustainable planning (e.g., traffic planning, waste management, recreation, and climate adaptation). In other words, while the focus on ecosystem services was non-negotiable, municipalities and researchers could adapt its application to their needs and interests. Some municipalities were already using ecosystem services in planning, both implicitly (e.g., Helsingborg) and explicitly (e.g., Lomma), while for others, it was new and/or difficult to understand.

Criteria for the project’s success were outlined in the research proposal, namely: popular science reports; recommendations and guidelines for ecosystem services planning for municipal use; a web-based knowledge node on SALA’s website; scientific publications; and presentations at international conferences (Jönsson et al. 2013). The importance of societal ‘products’ was repeatedly emphasised by municipal representatives during project workshops and meetings.

Once the project was underway, it became apparent that participants were divided over whether the research objective was to simply analyse, or also to promote the implementation of the ecosystem services concept in municipal planning. While most participants agreed on the importance of nature, and the need to safeguard and support ecosystem services in the development process, the initial research questions were formulated as neutral descriptive–analytical questions, such as “What are the main structural obstacles to implementing the ecosystem services approach in municipal planning?” (Jönsson et al. 2013, p. 1). However, during phase B, the collaborative process with municipalities partly led the research to evolve in a more normative direction, with the underlying assumption emerging that ecosystem services implementation was the desired orientation (see Box 2).

### A4. Methodological framework

- **Guiding questions:** Did the project team agree upon a jointly developed methodological framework that defined how the research target should be pursued in phase B and what transdisciplinary settings should be employed? Did the project organisation adequately account for both collaboration among scientific fields and how/whether researchers should collaborate with the municipal representatives in between workshops?
- **Attainment:** Low-Medium

Two factors largely influenced the organisation of work in the original research proposal. The first was the aim, derived from the initial survey of municipalities, to assess past decisions, current planning, and future challenges for ecosystem services planning. The second was each researcher’s area of skills and expertise, which resulted in the division into subprojects (Table 2). While this partitioned the work of researchers, it did not provide sufficient guidance for collaborative knowledge production and integration, especially with regard to the roles of, and collaboration with, municipality representatives. In most cases, it was left to subproject research teams to design their own framework for collaboration with the respective municipal representative(s). The exception was the analysis of municipality-specific case studies (subproject F, Table 1), in which a strong link between municipalities and local university campuses was planned and explicitly discussed with all participants. Full consensus on the specific methodology and methods for data collection and analysis to be used in each subproject was not possible due to the size, timeframe, and the disciplinary range of the project. A structured reintegration of the findings from the different subprojects (such as comparing and contrasting findings obtained through different methods, see, e.g., Greene et al. 1989) was also not planned for, which may have been a barrier to epistemic integration in later stages. However, the general organisation and development of work were continuously discussed and, if needed, modified (see “B2. Apply and adjust integrative research methods and transdisciplinary settings”).

The most important setting for collaboration between researchers and municipality representatives was the biannual (spring and autumn) workshops that took place in conference centres in partner municipalities. The aim of the workshops was to create cohesion and build shared ownership through mixed collaborative working sessions, shared meals and social activities such as nature walks. The final division of work and methods applied are shown in Table 2.

### B1. Roles of practitioners and researchers

- **Guiding question:** Were the tasks and roles of the involved researchers and municipality representatives clearly defined?
- **Attainment:** Low-Medium

Researchers were largely responsible for planning, carrying out, synthesising and reporting back from the different subprojects, especially as project funding did not cover municipality representatives’ time and involvement. The latter was identified as a key barrier and resulted in various discussions between participants at the outset of the project. The project leader was a university-based researcher; he was responsible for coordination, facilitation of workshops, and presenting the project’s outcomes to the funding agency. Municipality representatives acted as planning experts, providing key insights about the municipal organisation and processes and working to ensure that the knowledge produced was relevant and useable in practice. This did not always function smoothly in practice. One municipal representative stated, when asked about collaboration outside the workshops, “researchers have requested my expertise in municipal and comprehensive planning, but so far, it has rather been a question of data collection.” A project steering group, including two municipality representatives, regularly discussed the research focus and activities. However, as the project unfolded, it became apparent that neither researchers nor municipality representatives were completely sure of their role and involvement in the project. Although overall participants were satisfied with the process, in some cases, expectations were not fulfilled. At the same time, it also provided leeway for unexpected collaborations and participants taking on new roles to contribute to the project’s goals (see Box 3).

### B2. Apply and adjust integrative research methods and transdisciplinary settings

- **Guiding questions:** Did the team employ or develop suitable settings for inter- and transdisciplinary cooperation and knowledge integration? Did the research team employ or develop methods suitable to generate solution options to address the lack of knowledge and tools for consideration of ecosystem services in municipal planning and climate adaptation?
- **Attainment:** Medium

Regular workshops and case-based collaboration were the two key features designed to support inter- and transdisciplinary integration. For geographically focused cases, local university campuses in Kristianstad, Malmö, and Helsingborg were seen as natural platforms for integration between researchers and municipality representatives. Eventually, the ongoing dialogue fostered the establishment of new, problem-based case studies in the smaller municipalities that did not have their own university campus (such as Trelleborg and Lomma, see Box 3). Other case studies (e.g., Malmö) were partly abandoned due to lack of participation of municipal staff, which was related to the lack of funding (see “B1. Roles of practitioners and researchers”).

While joint workshops were essential to understand each other’s work, they also highlighted differences in preferred methods and approaches to participatory research. For instance, different scientific paradigms or worldviews led to contrasting views on the scientific validity of using workshop discussions to collect/create data, rather than conventional, ‘objective’ quantitative methods. Consequently, what characterised a ‘suitable’ method by scientific standards was continuously discussed.

Trade-offs were also perceived between fulfilling societal and scientific criteria. As one researcher noted in the questionnaire, “Sometimes the discussions about municipal work are on a too-detailed level, which can take focus off the scientific work.” In another case, a municipal representative used his/her participation in the project to instigate real-world change by suggesting specific civil servants and politicians for an interview study, based on whom (s)he thought needed to be made more aware of ecosystem services. The latter led to a discussion of societal relevance versus traditional scientific rigour.

The suitability of methods to generate solution options was regularly discussed, together with potential conflicts with researchers’ focus on producing scientific results. These discussions led, for instance, to a reduced focus on the six-step model from The Economics of Ecosystems and Biodiversity (TEEB 2010), which had been emphasised in the project application (Jönsson et al. 2013). During workshops, municipal representatives said that they considered it partly inherent in the Swedish planning process and therefore of little relevance. The initial research question based on the TEEB model was, therefore, replaced by one on the participatory working format, which was of common interest.

### C1. Two-dimensional integration

- **Guiding questions:** Have the project’s results been implemented to resolve or mitigate the lack of knowledge and tools for ecosystem service consideration in municipal planning? Were the results integrated into the existing scientific body of knowledge for transfer and scaling-up efforts?
- **Attainment:** Medium-High

There is still no full picture of how ECOSIMP’s outcomes will be received and applied. However, municipal representatives stated that the project has led to increased knowledge and awareness, and influenced municipal engagement in other topic-related projects. In particular, the project has contributed to the development of the Coastal Adaptation Strategy for Lomma (see Box 3), and a Coastal and Marine Plan in Kristianstad, both of which are expected to be adopted in 2017. In Trelleborg, ecosystem services are now considered in the ongoing planning of a ring road in the Dalköpinge river area and in the development of a new municipal strategy for stormwater management. “ECOSIMP has made a difference; ecosystem services, and especially cultural ecosystem services, now have a place in the discussions,” noted the municipal representative.

The national and regional contexts from which ECOSIMP emerged, i.e., the national-level Interim Target, SALA’s regional network activities, and the related ‘research municipality’ certification (see “The ECOSIMP project—case description and rationable”), provided favourable conditions for the transfer and scaling-up of the results in the policy domain. Results from the seven research projects funded by SEPA under the ‘Value of ecosystem services’ initiative were reported at a final conference in March 2017. They form a basis for how SEPA moves forward in ecosystem-based planning and development.^3^ Likewise, SALA considered ECOSIMP a pilot project in the Scania region, and experiences and lessons from the project are expected to feed into the organisation’s ongoing boundary work.

Finally, whereas the project’s scientific impact is still unknown, its various publications have fed into academic and operational debates on transdisciplinarity, urban planning, ecosystem services, and ecosystem-based adaptation. Regarding the latter, cooperation and outcomes have been partly up-scaled and applied in the German context (Wamsler 2015a; Wamsler and Pauleit, 2016).

### C2. Targeted ‘products’ for both parties

- **Guiding questions:** Did ECOSIMP provide Swedish-language tools, guidelines, pamphlets, reports, or other products useful for municipalities? Were peer-reviewed articles, book chapters and conference presentations generated in Swedish and English?
- **Attainment:** High

While there was a focus on scientific articles in international journals as the main measure of productivity, other outputs were generated in both English and Swedish. These included a final, practice-oriented report to SEPA (Jönsson et al. 2017), a booklet for municipalities on the project’s results and recommendations (2017, in preparation), case-specific reports (Bramryd et al. 2016; Bramryd and Johansson 2016), a popular science book chapter (Schubert et al. 2017b), and popular online articles (Hållbarhetsforum 2015a, 2015b). Planning guidelines for implementing (ecosystem-based) climate adaptation into municipal work (Wamsler 2015b; Wamsler and Brink 2016) were also developed and tested. Swedish resources will be published on SALA’s website.

Products for academia took the form of journal articles (e.g., Beery et al. 2016; Wamsler 2015a; Wamsler et al. 2014) and conference presentations and papers (Ekelund et al. 2015; Palo 2015). During the course of the project, encouraging co-authorship of articles across subprojects was found to increase internal review, feeling of ownership, and personal incentives among researchers. In addition, the prevailing assumption that municipality representatives were not interested in co-authoring scientific publications did not hold. Some municipality representatives highlighted the lack of opportunities for trained researchers working outside academia to build their reputation through the publication of journal articles. Although seen as positive for transdisciplinarity, extended use of co-authorship may challenge publication ethics guidelines, which require authors’ “substantial contribution” (Graf et al. 2007, p. 3) to, on one hand, the paper idea or data collection/analysis, and, on the other hand, writing or critical revision. In particular, researchers faced a dilemma concerning the invitation of municipal representatives as co-authors, since they (corresponding to the first criteria above) contributed to rich data and its interpretation throughout the three-year project, but (corresponding to the second criteria) expressed (or, in some cases, researchers assumed) that they had limited time to read and write scientific manuscripts.

### C3. Evaluate scientific and societal impact

- **Guiding questions:** Were the goals achieved? What additional (unanticipated) positive effects were created?
- **Attainment:** Medium

At the present time, all of the research questions have been addressed, and the criteria for success given on the original application have been, or are being, fulfilled. However, some of the societal actors had higher expectations, and hoped that the project would result in more practical guidance for municipalities. This issue was also raised by an external reviewer of the final report (see “X1. Continuous formative evaluation”). There may be several reasons for this mismatch in expectations and outcomes. First, there was little explicit discussion and revision of the criteria for success (including at the subproject level) once the project had been funded. Second, the project was not designed around the Lang et al. (2012) model of transdisciplinarity (Fig. 1). It was primarily funded as a scientific research project, and societal products (while specified in the funding application) were sometimes conceived as an automatic by-product of the scientific outcomes and related practitioner involvement. Accordingly, personal responsibilities were not as clearly assigned for societal products as for scientific studies. Third, the municipal co-funding model restricted the time municipality representatives could give to the project, which meant that although they possessed crucial competences, they were limited to providing input rather than taking an active part in the development of societal products (see “X3. Capabilities for and interest in participation”). The case study on Lomma’s Coastal Adaptation Strategy is an exception and a positive, unanticipated outcome (Box 3).

Another unanticipated positive outcome is the study presented in this paper. Analysing transdisciplinarity was not a part of the original project plan (with the call from the Swedish Environmental Protection Agency focused on ecosystem services knowledge development). However, as time went by, the participants became increasingly interested in the transdisciplinary study; it eventually became a formal subproject in the project organisation and attracted positive attention from the funding agency.

Other continuous, or interim, effects were observed throughout the project’s lifetime. Municipality participants, for example, noted that they could make use of the project to increase general knowledge and learning among their colleagues and local politicians. A municipal ecologist stated in the questionnaire: “the mere fact that we participate in a government mandate [governmental financed project] means that planners listen more and read up on [the ecosystem services concept] and bring it into the planning process.” Representatives from municipalities with established ecosystem services planning processes tended to emphasise the opportunity to concretise and receive feedback on their ideas, link their practice to theory, and increase the legitimacy of, and disseminate knowledge on their ongoing projects and plans. Conversely, the project provided researchers with invaluable insights into actual practice. One researcher stated: “I have attained a much greater awareness of how municipalities work and which aspects are important [for ecosystem services implementation]. Our long meetings have contributed to deep knowledge about this, as opposed to shallow or speculative knowledge.” Other positive effects were the opportunity for young researchers to gain confidence and experience through repeated meetings with the project group, including opportunities to collaborate with practitioners and gain support from senior researchers. Both researchers and municipality representatives emphasised that the project had created an extended network for potential future cooperation. Systematic tracking of future outcomes, however, will be a challenge, especially as there is no funding in place.

### X1. Continuous formative evaluation

- **Guiding question:** Has formative evaluation been conducted throughout the project, involving relevant experts on the empirical content (ecosystem services, climate adaptation and municipal planning) as well as transdisciplinarity?
- **Attainment:** Medium

As regards the project’s empirical content, the regular meetings and workshops, to which other practitioners and experts were invited, were an important mechanism for reflexivity and formative evaluation. Furthermore, evaluation by, and consultation with, municipality representatives was ongoing throughout each subproject. External peer review was provided via project participants’ presentations in international conferences, other events arranged by SALA or municipalities outside the Scania region, as well as the up-scaling and testing of some research outcomes in other contexts (see Wamsler 2015a). In addition, SEPA, the funding agency, provided its own review structure through its annual meetings and an anonymous expert panel review of the project’s final report. The review panel consisted of one natural and one social scientist (for the review of the scientific relevance) and a policy maker (for the review of the societal relevance).

In regard to transdisciplinarity, the study presented in this paper and the related workshop sessions were the main vehicle for formative evaluation. It benefitted from an internationally acknowledged framework (the design principles) and involved an external researcher with expertise on the topic. The transdisciplinary design principles and guiding questions were generally well received by the participants, especially the municipality representatives, since they allowed a more objective discussion of issues that could otherwise have been perceived as too political or sensitive, and thus facilitated new voices being heard in the project. However, some circumstances limited or delayed corrective action. First, the principles were introduced mid-way, in a bottom–up manner by a PhD student, rather than prescribed by the steering group. Second, while the steering group was positive to such follow-up of the working method, achieving transdisciplinarity was not an explicit success criterion (and related efforts thus competed with achieving the project’s content-related goals).

### X2. Mitigating conflict constellations

- **Guiding question:** Did the researchers and municipality representatives prepare for/anticipate conflict at the outset, and were procedures/processes adopted for managing conflict as and when it arose?
- **Attainment:** Low-Medium

No open conflicts occurred. This is probably due to participants’ shared values regarding environmental management in general, their interest in problem-based research and work, and continuous attempts to adapt to different constraints and demands. In addition, the project workshops included social activities to strengthen cohesion and joint ownership between and among municipality representatives and researchers. Although no explicit procedures or processes were designed for managing potential conflicts, this helped to deal with the different challenges that arose (as discussed in “B2. Apply and adjust integrative research methods and transdisciplinary settings”, “C2. Targeted ‘products’ for both parties”, and “C3. Evaluate scientific and societal impact”). An issue that was identified early on was the researchers’ wish to provide municipality representatives with useful information while preserving scientific integrity and ethics. For instance, should interview data from interviews with citizens and (subordinate) colleagues be shared with municipal collaborators, given that they are in a power relation vis-a-vis these groups? Because research-related challenges were openly discussed and social activities helped to strengthen cohesion and ownership, such potential conflicts could be mitigated.

### X3. Capabilities for and interest in participation

- **Guiding question:** Has adequate attention been paid to the (material and intellectual) capabilities required for effective and sustained participation in the project over time?
- **Attainment:** Medium

There was generally a high (material and intellectual) capacity to participate in the project. Meetings were held in different locations in partner municipalities at times that suited those who had to travel. Many participants were accustomed to acting at the interface of research and practice (e.g., networks, conferences, and boundary organisations) and had a strong personal interest in the topic. The fact that some municipality representatives were trained researchers also helped to strengthen capacities; in fact, it proved to be a key factor for their sustained engagement. Furthermore, networking and cooperation among participating municipalities were identified as an important motivating factor and a potential driver for municipalities to participate in research projects.

However, relations external to the project, such as the roles of planners and ecologists in their own municipalities, were found to affect their ability to act, especially in terms of how much time they could devote to the project’s activities. It became obvious in workshop discussions that many municipality representatives had to continually justify their participation to their superiors, and were required to prioritise issues that were more urgent. This highlights an important barrier to municipalities’ participation. Hours worked on the project were co-funded by the municipality, and this arrangement proved to be too restrictive or insufficiently specified in the project plan. Consequently, they had little time to devote to the project in between official meetings and workshops, and were often not replaced when their responsibilities changed or a short-term contract ended (see “B2. Apply and adjust integrative research methods and transdisciplinary settings”). The result was a decrease in municipality representatives as the project unfolded, while the number of researchers increased (e.g., due to the involvement of new PhD students). Municipal staff mentioned the lack of access to scientific input (journals, seminars) as another barrier to their engagement in research cooperation.

**Box 3:**
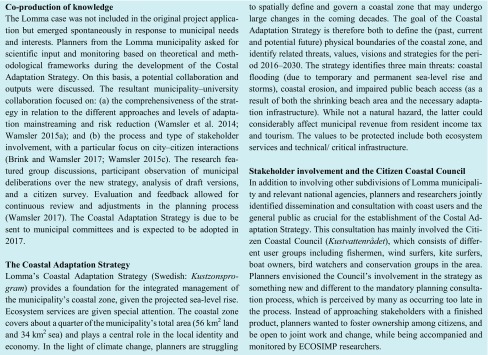
Lomma case: Inclusive adaption planning for a changing coastal zone

## Discussion

Increasing interaction between research and governance work in urban sustainability requires attention to related challenges and how they can be addressed. Based on our assessment of the ECOSIMP project, in this section, we discuss its implications for university–municipality collaboration and reflect on the use of the applied analytical framework. We hereby present five key messages, numbered consecutively throughout the next two subsections.

### Opportunities and challenges on the road to ‘research municipalities’

#### Lesson 1: More attention to the purpose of stakeholder participation can justify their selection and roles

The selection of societal actors for transdisciplinary projects can be subject to scrutiny on a number of fronts, including scientific methods, functionality, and democratic/representative perspectives. Transdisciplinary design principles recommend the inclusion of all relevant expertise, experience and other ‘stakes’ needed to create sustainable solution options and contribute to science (Lang et al. 2012). In this context, the selection of municipal civil servants as a key, non-academic actor in ECOSIMP can be challenged. Despite their key roles for ecosystem services implementation, they did not represent a wide and diverse number of societal groups or interests and had limited power to establish and drive long-term change. In addition, as described in “B1. Roles of practitioners and researchers”, participants were sometimes unsure about their role in the project themselves. The design principles call into question, but do not provide sufficient guidance on whether the project should have included additional stakeholders. Lang et al. (2012) argue, on one hand, for a sufficient number and diversity of stakeholders with a legitimate stake, but admit, on the other hand, that limited resources and methodological reasons often lead to a relatively small number of participants, or the network of ‘usual suspects’.

A potentially useful contribution comes from Renn and Schweizer (2009) who show how the different theoretical traditions underlying knowledge on participation affect practical choices such as the number of participants, the need for consensus, and participation method (e.g., focus groups, internet-based participation, citizen forums, workshops, or panel discussions). They describe, for instance, how *functionalist* approaches to participation focus on improving decision output by including relevant knowledge carriers in relation with a predefined problem; *deliberative* participation aims to create a legitimate decision-making process that reflects social and cultural values by including a diversity of views and reaching a consensus through argumentation, while the *neo-liberal* model focuses on representing all values and preferences in proportion to their share in the affected group and thereby seeks a solution that optimises the payoffs for each stakeholder. Yet another tradition, *transformative* (or emancipatory) participation, focuses on building strategies to empower marginalised groups (Renn and Schweizer 2009). In the latter, (only) representing the marginalised perspective is said to contribute to so-called “strong objectivity” (Rosendahl et al. 2015, p. 17).

According to these categories, stakeholder involvement in ECOSIMP can be interpreted as both functional and transformative, i.e., aiming to include highly knowledgeable societal actors in the strategic development of an issue that was seen as ‘marginalised’ in the municipal organisation (relative to economic growth). These two perspectives may be incompatible, since functionalism has been criticised for over-emphasising the beneficial effects of institutional structures and prioritising social control over social change, thus promoting incremental, or adjustive, rather than transformative change (Wallace and Wolf 2005). Still, these categories highlight that more stakeholders, with more diverse views, are not always better with regard to the goals of the project. Rather, in determining who has a legitimate stake, transdisciplinary project design needs to consider, not only who influences or is influenced by the ‘real-world’ problem, but the specific purpose and theoretical underpinnings of stakeholder participation in the research process.

#### Lesson 2: Concrete and interim benefits for societal participants need to be continuously discussed

Whereas power asymmetries based on social categories have been highlighted in the literature as a potential challenge to transdisciplinary projects (Jahn et al. 2012; Mobjörk 2010; Wittmayer and Schäpke 2014), this was not perceived as a key concern in the ECOSIMP team, which consisted of a homogeneous, highly educated group of professionals and experts. However, small asymmetries arising from the requirements and responsibilities of the different job categories were identified, such as in the level of influence on, and the rewards gained from the project. This is consistent with the idea that “planning is not science, but [it is rather] social action with scientific, technological and legal underpinnings” (Ahern et al. 2014, p. 255). Consequently, actors from municipalities were more directly accountable to their superiors, politicians, and citizens than researchers. Institutional structures (notably, the research funding structure) further increased asymmetries, as researchers had no responsibility to follow-up on long-term impacts. In this context, Russel et al. (2008) problematise how the outcomes of mutual learning processes can remain at the theoretical level and/or end up as intellectual property of researchers in the form of academic articles.

The fact that transdisciplinary work is (increasingly) rewarded and reinforced in the research community, but not fully supported in associated funding structures and the municipal government context must be acknowledged and addressed. Our results indicate that if societal actors’ motivation to participate in transdisciplinary collaboration is to increase, the concrete and interim benefits for both participating individuals and their organisations need to be strengthened. In addition, practitioners need to be able to devote sufficient time. This may require political reorganisation, procuring external funding, allocating more hours than the time it takes to attend physical meetings, securing replacements for municipality representatives if they leave, and financing long-term implementation and follow-up.

#### Lesson 3: Promoting ‘inter’ can support transdisciplinarity

While bridging the science–policy gap is often a priority in transdisciplinary projects, our findings demonstrate that such projects are equally a space for interdisciplinary and (in the case of urban governance research) inter-city learning. In ECOSIMP, municipalities were keen to learn about local examples of ecosystem services and adaptation planning, and promote success stories. This is in line with other research that has shown the importance of inter-city networks for both capacity building and municipal branding (Busch 2015). Inter-city learning can help to address the limited transferability of outcomes (e.g., overly specific case-based solution strategies), which is a key challenge for transdisciplinarity (Lang et al. 2012). For instance, while the discussions in ECOSIMP created an understanding of general focal points and hurdles for ecosystem services implementation in the Swedish planning system, they also uncovered differences between the municipalities’ internal structures and processes.

Meanwhile, difficulties relating to interdisciplinary processes should not be under-estimated. Most importantly, the ontological, epistemological, and theoretical assumptions that underlie different research approaches, as well as the competence to navigate related conflicts, require explicit consideration in the project’s design and implementation. Acceptance of and trust in other project members’ disciplinary expertise are also crucial.

### Reflection on the analytical transdisciplinarity framework

#### Lesson 4: Design principles can mitigate project shortcomings, even when transdisciplinarity is not an explicit aim

While the purpose of this assessment was not to judge how well ECOSIMP delivered on the project aims, it has allowed us to identify areas, where essential features of transdisciplinarity (i.e., what the design principles prescribe) were lacking and tentatively connect them to the main weaknesses in the project’s outcomes (i.e., what it set out to do). A major self-criticism is that there could have been more focus on societal products to meet participants’ expectations. Here, the initial structuring of the project around the Lang et al. (2012) model (Fig. 1), which depicts transdisciplinarity as a process with two epistemic ends (rather than seeing societal outcomes as by-products of scientific outcomes), could have enhanced the importance given to the societal products, including the allocation of time and assigning responsibilities. In addition, a more explicit discussion of success criteria, as recommended by the framework (principle A3), would have been useful. What different participants define as success, however, may evolve throughout the project as they adapt to limitations or identify opportunities for mutual gain through repeated dialogue (Ansell and Gash 2008).

A second criticism arising from the self-assessment is that the different subprojects (and related disciplines) remained quite disconnected in the project’s outcomes (e.g., as separate chapters in the final report). While there was a relatively high level of social-organisational integration (goals of actors and subprojects) and communicative integration (common language), epistemic integration could have been stronger. Structuring the work around a design concept or framework (as recommended by principle A4; e.g., lifecycle approach of a pharmaceutical product in Jahn et al., 2012) could have facilitated knowledge integration in the final phase. In this context, the explicit use of the ecosystem service concept or framework for epistemic integration deserves more attention in transdisciplinary scholarship.

On this basis, our ex-post assessment indicates that the project could have benefitted from using the Lang et al. (2012) principles from the outset. Since they were unfamiliar to participants at the time of project initiation, and later introduced in a bottom–up manner, they had limited effect (see “X1. Continuous formative evaluation”). Application of transdisciplinary design principles in similar projects could perhaps be increased through their adaptation and publication in different languages, their recommendation by funding agencies in sustainability-related topics alongside their requirements for stakeholder involvement, and by researchers and practitioners gaining more transdisciplinary competence and experience.

#### Lesson 5: Design principles can allow new voices to be heard

While the participants’ reaction to the transdisciplinary assessment was generally positive, there were some differences. As described in “X1. Continuous formative evaluation”, the practitioners welcomed the meta-analysis, since it facilitated an objective discussion of challenges in the project and further allowed for the joint workshop discussions to be captured in a way that was not possible through ‘objective’ natural science methods. The researchers were more hesitant of how critical one could be towards the project (and each other’s work) and there were methodological differences regarding the use of workshop discussion and observation as data. However, framing the self-assessment as a joint paper that could contribute to science, as well as positive reactions from reviewers and funding agency, helped encourage an atmosphere of constructive criticism in this regard.

Although the design principles and guiding questions call the project’s transdisciplinarity into question, they generally served to assess and analyse the project. In particular, the principles helped to guide through the complexity and focus on the general project level while being flexible enough to include emerging considerations. For instance, under the societal and scientific products principle (C2), the issue of ownership over intellectual property (i.e., co-authorship on scientific papers) could be raised.

Based on our experience, we formulate the following additional guiding questions (with the associated project phase/design principle shown in parenthesis), which can help inspire reflexivity and strengthen the aspects outlined in the discussion in similar projects:

- Does the project group agree on the purpose of involving societal actors in the research? (e.g., functional, deliberative, emancipatory) (A1 or B1)
- Do researchers clearly articulate their ontological and epistemological positions/research paradigms and does the project group discuss or agree on the implications for conducting and synthesising the research? (A4)
- Does the project group agree on a strategy for ethical data management (e.g., to protect third-party informants who may be in a position of dependency on the project’s societal participants)? (A4 or X2)
- Do the participants make explicit what additional or interim rewards (apart from official success criteria) that they or their organisation could get from their participation (e.g., career benefits, contact with thesis students)? (B1 or X3)
- Does the group agree on a strategy for giving credit to participants for the intellectual property resulting from the work (e.g., tools and publications)? (C2)
- Do participants agree on how the different methodological approaches complement each other (triangulation, complementary, development, etc.; see the literature on mixed methods, e.g., Greene et al. 1989)? (B2)
- Do the findings from different research perspectives (e.g., quantitative and qualitative) converge? (C3)

## Conclusion

Transdisciplinary processes that promote sustainable urban transformations have received greater attention in recent years. The ambitions and methods used in these endeavours will continue to diversify. The assessment of both successes and failures is thus warranted.

Against this background, the aim of this study was to appraise a research project focused on supporting ecosystem services and adaptation planning in seven municipalities in southern Sweden. Based on a participatory assessment along principles of transdisciplinarity, our results show how the management of university–municipality collaborations can be improved. In particular, we argue that: (1) selecting the number and type of project stakeholders requires more explicit consideration of the purpose of societal actors’ participation; (2) concrete, interim benefits for participating practitioners, and organisations need to be continuously discussed; and (3) facilitating interdisciplinary and inter-city learning and collaboration are key factors that support transdisciplinarity and, ultimately, urban sustainability and long-term change. In this context, we conclude that design principles and guiding questions for transdisciplinarity have the potential to (4) mitigate project shortcomings, even when transdisciplinarity is not an explicit aim, and (5) address differences and allow new voices to be heard. Based on the assessment, we propose additional guiding questions that can address shortcomings and inspire reflexivity in transdisciplinary projects.

## Footnotes

1 While the term ‘participatory’ can be linked to particular literatures and approaches (e.g., Glassman and Erdem 2014; Johansson and Isgren 2017; Streck 2014), we use it here in its general sense to denote research collaboration with actors outside academia, as opposed to ‘transdisciplinary’, which we reserve for the approach described in Lang et al. (2012).

2 These meetings brought together the same project participants at regular intervals. Minor differences in attendance were due to each participant’s availability.

3
http://ecosystemservices.se/slutkonferens.4.6a63a18158efefeeb9ff.html (in Swedish).

## References

[CR1] Abson DJ, von Wehrden H, Baumgärtner S, Fischer J, Hanspach J, Härdtle W, Heinrichs H, Klein AM, Lang DJ, Martens P, Walmsley D (2014). Ecosystem services as a boundary object for sustainability. Ecol Econ.

[CR2] Adger WN, Quinn T, Lorenzoni I, Murphy C, Sweeney J (2013). Changing social contracts in climate-change adaptation. Nat Clim Change.

[CR3] Ahern J, Cilliers S, Niemelä J (2014). The concept of ecosystem services in adaptive urban planning and design: a framework for supporting innovation. Landsc Urban Plan.

[CR4] Ansell C, Gash A (2008). Collaborative governance in theory and practice. J. Public Adm Res Theory.

[CR5] Beery T, Stålhammar S, Jönsson KI, Wamsler C, Bramryd T, Brink E, Ekelund N, Johansson M, Palo T, Schubert P (2016). Perceptions of the ecosystem services concept: opportunities and challenges in the Swedish municipal context. Ecosyst Serv.

[CR6] Borgström S (2013) Ekosystemtjänstperspektivet i svensk miljöpolicy och praktik – potentialer, barriärer och vägar mot integration. En översiktlig sammanfattning av ett pågående forskningsprojekt vid Stockholm Resilience Centre, Stockholms Universitet

[CR7] Bramryd T, Johansson M (2016). Analys av Ekosystemtjänster inom tätortsnära grönområden i Båstad.

[CR8] Bramryd T, Johansson M, Eriksson A (2016). Görarpsdammens ekosystemtjänster i Rååns vattensystem.

[CR9] Brandt P, Ernst A, Gralla F, Luederitz C, Lang DJ, Newig J, Reinert F, Abson DJ, von Wehrden H (2013). A review of transdisciplinary research in sustainability science. Ecol Econ.

[CR10] Brink E, Wamsler C (2017). Collaborative governance for climate change adaptation: mapping citizen–municipality interactions. Enviro Policy Gov.

[CR11] Bulkeley H, Betsill M (2005). Rethinking sustainable cities: multilevel governance and the “Urban” politics of climate change. Environ Polit.

[CR12] Bunders PDJFG, Broerse DJEW, Keil DF, Pohl DC, Scholz PDRW, Zweekhorst DMBM, Veld RJ (2010). How can transdisciplinary research contribute to knowledge democracy?. Knowledge democracy.

[CR13] Busch H (2015). Linked for action? An analysis of transnational municipal climate networks in Germany. Int J Urban Sustain Dev.

[CR86] Chan KMA, Shaw MR, Cameron DR, Underwood EC, Daily GC, Reid W (2006). Conservation planning for ecosystem services. PLoS Biol.

[CR14] Crabtree BF, Miller WL (1999). Doing qualitative research.

[CR15] Daily G (1997). Nature’s services: societal dependence on natural ecosystems.

[CR16] Ekelund N, Schubert P, Roth A, Bramryd T, Jönsson KI, Wamsler C, Johansson M, Beery TH, Stålhammar S, Brink E, Palo T (2015) Trends in municipal planning and comprehensive plans (1980-2014) from an Ecosystem Services Approach in south Sweden. Presented at the 8th ESP World conference: ecosystem services for nature, people and prosperity, Stellenbosch, South Africa

[CR17] Ernstson H, Barthel S, Andersson E, Borgström ST (2010). Scale-crossing brokers and network governance of urban ecosystem services: the case of Stockholm. Ecol Soc.

[CR18] Feola G (2014) Societal transformation in response to global environmental change: a review of emerging concepts. AMBIO, pp 1–15. doi:10.1007/s13280-014-0582-z 10.1007/s13280-014-0582-z PMC4510318 25431335

[CR19] Funtowicz SO, Ravetz JR (1993). Science for the post-normal age. Futures.

[CR20] Glassman M, Erdem G (2014). Participatory action research and its meanings Vivencia, Praxis, Conscientization. Adult Educ Q.

[CR21] Gómez-Baggethun E, Barton DN (2013). Classifying and valuing ecosystem services for urban planning. Ecol Econ.

[CR22] Graf C, Wager E, Bowman A, Fiack S, Scott-Lichter D, Robinson A (2007). Best practice guidelines on publication ethics: a publisher’s perspective. Int J Clin Pract Suppl.

[CR23] Greene JC, Caracelli VJ, Graham WF (1989). Toward a conceptual framework for mixed-method evaluation designs. Educ Eval Policy Anal.

[CR24] Hållbarhetsforum (2015a) Nytt verktyg för kommuners klimatanpassning (Nina Nordh) [WWW Document]. Hållbarhetsforum Vid Lunds Univ. URL http://www.hallbarhet.lu.se/article/nytt-verktyg-for-kommuners-klimatanpassning. Accessed 20 April 2016

[CR25] Hållbarhetsforum (2015b) Klimatanpassning i fokus i Lomma kommun (Nina Nordh) [WWW Document]. Hållbarhetsforum Vid Lunds Univ. URL http://www.hallbarhet.lu.se/article/klimatanpassning-i-fokus-i-lomma-kommun. Accessed 20 April 2016)

[CR26] Hirsch Hadorn G, Pohl C (2007). Principles for Designing Transdisciplinary Research.

[CR27] Hirsch Hadorn G, Bradley D, Pohl C, Rist S, Wiesmann U (2006). Implications of transdisciplinarity for sustainability research. Ecol Econ.

[CR28] IPCC (2014) Annex II: Glossary. In: Barros VR, Field CB, Dokken DJ, Mastrandrea MD, Mach KJ, Bilir TE, Chatterjee M, Ebi KL, Estrada YO, Genova RC, Girma B, Kissel ES, Levy AN, MacCracken S, Mastrandrea PR, White LL (eds) Climate change 2014: impacts, adaptation, and vulnerability. Part B: Regional aspects. Contribution of working group II to the fifth assessment report of the intergovernmental panel on climate change. Cambridge University Press, Cambridge, UK, New York, NY, USA, pp 1757–1776

[CR29] Jahn T, Bergmann M, Keil F (2012). Transdisciplinarity: between mainstreaming and marginalization. Ecol Econ.

[CR30] Johansson E, Isgren E (2017) Local perceptions of land-use change: using participatory art to reveal direct and indirect socioenvironmental effects of land acquisitions in Kilombero Valley, Tanzania. Ecol Soc 22. doi:10.5751/ES-08986-220103

[CR31] Jönsson KI, Palo T, Ekelund NGA, Bramryd T, Wamsler C (2013). Implementing the ecosystem services approach at the municipal level—a transdisciplinary project with coastal communities in south Sweden (funding proposal).

[CR32] Jönsson KI, Ekelund NGA, Wamsler C, Brink E, Beery TH, Palo T, Schubert P, Stålhammar S, Bramryd T, Johansson M (2017). Implementering av ekosystemtjänstbegreppet i kommunal verksamhet—Slutrapport.

[CR33] Lagercrantz K, Palo T (2012). Forskningsbehov hos Skånes kommuner inom miljö och samhällsbyggnad: Analyser av intervjuer, tankesmedjor och en workshop (text).

[CR34] Lang D, Wiek A, Bergmann M, Stauffacher M, Martens P, Moll P, Swilling M, Thomas J (2012). Transdisciplinary research in sustainability science: practice, principles, and challenges. Sustain Sci.

[CR35] Luederitz C, Brink E, Gralla F, Hermelingmeier V, Meyer M, Niven L, Panzer L, Partelow S, Rau A-L, Sasaki R, Abson DJ, Lang DJ, Wamsler C, Von Wehrden H (2015). A review of urban ecosystem services: six challenges for future research. Ecosyst Serv.

[CR36] Max-Neef MA (2005). Foundations of transdisciplinarity. Ecol Econ.

[CR37] Mayring P (2000) Qualitative content analysis. Forum Qual Soc Res 1(2). doi:10.17169/fqs-1.2.1089

[CR38] McCauley DJ (2006). Selling out on nature. Nature.

[CR39] McCormick K, Anderberg S, Coenen L, Neij L (2013). Advancing sustainable urban transformation. J Clean Prod.

[CR40] Mobjörk M (2010). Consulting versus participatory transdisciplinarity: A refined classification of transdisciplinary research. Futures.

[CR41] Niemelä J, Saarela S-R, Söderman T, Kopperoinen L, Yli-Pelkonen V, Väre S, Kotze DJ (2010). Using the ecosystem services approach for better planning and conservation of urban green spaces: a Finland case study. Biodivers Conserv.

[CR42] Palo T (2013) Kommunernas klimatanpassningsarbete är att planera under osäkerhet. Intryck från en forskningscirkel i Kommunförbundet Skånes regi, april–juni 2013 (No. 7). Kommunförbundet Skåne, Lund

[CR44] Palo T (2015) The integration of concepts in municipality planning in relation to research interests, environmental concerns and policy decision tools. Presented at the 8th ESP World conference: ecosystem services for nature, people and prosperity, Stellenbosch, South Africa

[CR45] Palo T, Lagercrantz K, Bramryd T, Johansson M, Beery T, Jönsson K, Wamsler C, Brink E, Schubert P, Ekelund N (2016). Priority areas in municipality planning: ecosystem services, environmental impact assessments and research areas. One Ecosyst.

[CR46] Polk M (2014). Achieving the promise of transdisciplinarity: a critical exploration of the relationship between transdisciplinary research and societal problem solving. Sustain Sci.

[CR47] Renn O, Schweizer P-J (2009). Inclusive risk governance: concepts and application to environmental policy making. Environ Policy Gov.

[CR48] Roberts D (2008). Thinking globally, acting locally—institutionalizing climate change at the local government level in Durban, South Africa. Environ Urban.

[CR49] Rosendahl J, Zanella MA, Rist S, Weigelt J (2015). Scientists’ situated knowledge: Strong objectivity in transdisciplinarity. Futures.

[CR50] Russell AW, Wickson F, Carew AL (2008). Transdisciplinarity: context, contradictions and capacity. Futures.

[CR51] SALA (2016). Utanför det akademiska rummet: Forsknings- utvecklings- och innovationsmiljöer i praktiken.

[CR52] SALA (2016b) Forskningskommuner inom miljö och samhällsplanering [WWW Document]. Samhällsbyggnad Miljö Kult. Fritid Och Folk. URL http://kfsk.se/samhallsbyggnad/fou-miljo-och-samhallsbyggnad/forskningskommun/. Accessed 7 April 2016

[CR53] Scholz RW, Lang DJ, Wiek A, Walter AI, Stauffacher M (2006). Transdisciplinary case studies as a means of sustainability learning: historical framework and theory. Int J Sustain High Educ.

[CR81] Schubert P, Ekelund N, Beery T, Wamsler C, Jönsson I, Roth A, Stålhammar S, Bramryd T, Johansson M, Palo T (2017a) Implementation of the ecosystem services approach in Swedish municipal planning. Environ Policy Plan **(forthcoming)**

[CR54] Schubert P, Jönsson KI, Bramryd T, Johansson M, Brink E, Wamsler C, Palo T, Beery T, Ekelund N, Stålhammar S (2017b) Ekosystemtjänster—ett verktyg för en ny syn på utvecklingen mot ett hållbart samhälle. In: Urban utveckling och interaktion, Ymer SSAG, pp 213–237

[CR55] SEPA (2012). Sweden’s environmental objectives—an introduction.

[CR85] Staes J, Vrebos D, Meire P (2010) A framework for ecosystem services planning. In: Liotta PH, Kepner WG, Lancaster JM, Mouat DA (eds) Achieving environmental security: ecosystem services and human welfare. IOS Press, Amsterdam, pp 53–72

[CR56] Statskontoret (2016). Forskning i praktiken—om praktikrelevant forskning och praktiknära forskningsmiljöer.

[CR57] Streck DR (2014). Knowledge and transformative social action: the encounter of selected traditions of participatory (action) research. Glob Soc Educ.

[CR58] SymbioCity (2011). The Symbio City approach—conceptual framework for support to sustainable urban development in low and middle income countries.

[CR59] Talwar S, Wiek A, Robinson J (2011). User engagement in sustainability research. Sci Public Policy.

[CR60] TEEB (2010) The economics of ecosystems and biodiversity for local and regional policy makers. UNEP/Earthprint. http://www.teebweb.org/publication/teeb-for-local-and-regional-policy-makers-2/. Accessed 20 Oct 2017

[CR61] Turnhout E, Waterton C, Neves K, Buizer M (2013). Rethinking biodiversity: from goods and services to “living with”. Conserv Lett..

[CR62] Wallace RA, Wolf A (2005). Contemporary sociological theory: expanding the classical tradition,.

[CR63] Wamsler C (2015a) Mainstreaming ecosystem-based adaptation: transformation toward sustainability in urban governance and planning. Ecol Soc 20. doi:10.5751/ES-07489-200230

[CR64] Wamsler C (2015b) Guideline for integrating climate change adaptation into municipal planning and governance. Working Paper 31. Disaster Studies and Management Working Paper Series of the University College London (UCL) Hazard Centre. ECOSIMP/Lund University

[CR65] Wamsler C (2015). From risk governance to city–citizen collaboration: Capitalizing on individual adaptation to climate change. Environ Policy Gov.

[CR80] Wamsler C (2017). Stakeholder involvement in strategic adaptation planning: Transdisciplinarity and coproduction at stake?. Environ Sci Policy.

[CR66] Wamsler C, Brink E (2014). Interfacing citizens’ and institutions’ practice and responsibilities for climate change adaptation. Urban Clim.

[CR67] Wamsler C, Brink E (2016) Promoting nature-based solutions: a guideline for integrating ecosystem-based adaptation into municipal planning and governance. Working Paper 32. Disaster Studies and Management Working Paper Series of the University College London (UCL) Hazard Centre. ECOSIMP/Lund University

[CR68] Wamsler C, Pauleit S (2016) Making headway in climate policy mainstreaming and ecosystem-based adaptation: two pioneering countries, different pathways, one goal. Clim. Change 1–17. doi:10.1007/s10584-016-1660-y

[CR69] Wamsler C, Luederitz C, Brink E (2014). Local levers for change: mainstreaming ecosystem-based adaptation into municipal planning to foster sustainability transitions. Glob Environ Change.

[CR83] Wamsler C, Niven L, Beery TH, Bramryd T, Ekelund N, Jönsson KI, Osmani A, Palo T, Stålhammar S (2016) Operationalizing ecosystem-based adaptation: harnessing ecosystem services to buffer communities against climate change. Ecol Soc 21(1)

[CR70] Wiek A, Ness B, Schweizer-Ries P, Brand FS, Farioli F (2012). From complex systems analysis to transformational change: a comparative appraisal of sustainability science projects. Sustain Sci.

[CR71] Wittmayer JM, Schäpke N (2014). Action, research and participation: roles of researchers in sustainability transitions. Sustain Sci.

[CR72] Woodruff SC, BenDor TK (2016). Ecosystem services in urban planning: comparative paradigms and guidelines for high quality plans. Landsc Urban Plan..

[CR73] Yin RK (2008). Case study research: design and methods,.

